# Genome-Wide Association Analysis Reveals Key Genes Responsible for Egg Production of Lion Head Goose

**DOI:** 10.3389/fgene.2019.01391

**Published:** 2020-01-28

**Authors:** Qiqi Zhao, Junpeng Chen, Xinheng Zhang, Zhouyi Xu, Zhenping Lin, Hongxin Li, Wencheng Lin, Qingmei Xie

**Affiliations:** ^1^ College of Animal Science, South China Agricultural University and Guangdong Provincial Key Lab of Agro-Animal Genomics and Molecular Breeding, Guangzhou, China; ^2^ Key Laboratory of Animal Health Aquaculture and Environmental Control, Guangzhou, China; ^3^ Shantou Baisha Research Institute of Original Species of Poultry and Stock, Shantou, China

**Keywords:** lion head goose, genome wide association study, egg production, candidate genes, quantitative real-time polymerase chain reaction

## Abstract

The lion head goose is one of the most important agricultural resources in China; however, its breeding process is relatively slow. In the present study, a genome-wide association study was performed for the genetic selection of egg production characters in lion head geese. We detected 30 single-nucleotide polymorphisms located in or near 30 genes that might be associated with egg production character, and quantitative real-time polymerase chain reaction was used to verify their expression level in lion head geese. The results showed that the expression levels of *CRTC1* (encoding *CREB*-regulated transcription coactivator 1), *FAAH2* (encoding fatty acid amide hydrolase 2), *GPC3* (encoding glypican 3), and *SERPINC1* (encoding serpin family C member 1) in high egg production population were significantly lower than those in the low egg production populations (*P < 0.05). The expression levels of *CLPB* (encoding caseinolytic peptidase B protein homolog), *GNA12* (encoding guanine nucleotide-binding protein subunit alpha-12), and *ZMAT5* (encoding zinc finger, matrin type 5) in the high egg production population were significantly higher than those in the low egg production populations (*P < 0.05). The expression of *BMP4* (encoding bone morphogenetic protein 4), *FRMPD3* (encoding *FERM* and *PDZ* domain containing 3), *LIF* (encoding leukemia inhibitory factor), and *NFYC* (encoding nuclear transcription factor Y subunit gamma) in the high egg production population were very significantly lower than those in the low egg production population (**P < 0.01). Our findings provide an insight into the economic traits of lion head goose. These candidate genes might be valuable for future breeding improvement.

## Introduction

The lion head goose is named for the sarcoma that makes it resemble a lion's head from the front. Lion head geese provide great economic benefits via the widespread consumption of their meat as stewed products (Zhuang and Lin, 2006). Lion head geese, originating from Shantou Raoping in Guangdong province, are the only large and major goose species in China, and are the germplasm resources under special state protection (Chen et al., 2011). Lion head geese, whose ancestors are anser cygnoides, are herbivorous animals showing fast growth and large body size; as such, their feeding is relatively environment-friendly (Huang, 2009; He et al., 2012). However, they show low fertility with an average of 20–25 eggs per year (Zhang, 1991). The egg-laying period is not continuous but is divided into three parts caused by its strong broodiness (Wang, 2007). A lower laying rate may hinder the development of the lion head goose industry. Therefore, based on maintaining the characteristics of its original breeds, improving the low fecundity has become an important breeding objective of lion head goose, among which the egg laying characteristics are one of the most significant aspects (Wang et al., 2008). It is believed that egg production could be improved by adopting a modern genome-enhanced breeding scheme.

Genome-wide association studies (GWASs), which were proposed first by Risch in 1996, are powerful and effective tools to identify genetic markers associated with the trait of interest (Risch and Merikangas, 1996). In recent years, a large number of GWASs on human diseases have been published, such as for vitiligo (Shen et al., 2016) and for livestock animals such as pigs (Luo et al., 2012). Since the development of the HapMap Project, a number of high-density single-nucleotide polymorphism (SNP) chips for plant and animal species like chicken, swine, cattle, sheep, and the like have been developed as well (Gibbs et al., 2003). Hoglund et al. found that a total of 17,388 significant SNP markers and candidate genes associated with female fertility were distributed on 25 chromosomes in the Nordic Red cattle group (Hoglund et al., 2015). Shen *et al.* carried out GWAS on Ningdu Sanhuang chickens with Chicken chip and found the candidate gene, *GARNL1*, which was related to reproductive traits (Shen et al., 2012). Xie used the Illumina Porcine SNP60K chip to screen the potential candidate genes that may be associated with the litter size of the Xiang Pig (Xie, 2016). The production of SNP chips and the appearance of high-throughput sequencing technology have made GWAS an important research strategy in some fields. GWAS is widely accepted as a primary method for gene detection (Jiang et al., 2010).

In the present study, we performed GWAS to identify SNPs and potential genetic variants that may be associated with egg laying character of the lion head geese. Then we attempted to verify their functionality. As a result, we have identified certain genes that might play important roles in the egg laying process.

## Materials and Methods

### Animals Resources and Sample Collection

Lion head geese are the largest goose breed in China and are the one of the world's big goose species. In the past 2 years, we have bred a batch of lion head geese with high and low egg production in the Shantou Baisha Research Institute of Original Species of Poultry and Stock, Guangdong Province. These geese have the same growth environment and nutritional supplements, and they have free access to food and water.

A total of 217 geese blood samples were collected at the Shantou Baisha Research Institute of Original Species of Poultry and Stock, including 136 high egg-production geese (more than 35 eggs per year) and 81 low egg-production geese (less than 25 eggs per year). Blood samples were stored at an ACD anticoagulant tube at −80°C cryogenic refrigerator for further experiments.

### DNA Extraction and Whole Genome Sequencing

Genomic DNA was extracted from peripheral blood cells of the high and low egg production groups using a HiPure Blood DNA Mini Kit (Magenbio, Guangzhou, China). After passing the quality inspection of NanoDrop 2000 Spectrophotometer (Thermo, America), the DNA samples were sent to Beijing Genomics Institute (Shenzhen, China) for whole genome resequencing. An Easy DNA Library Prep Kit (MGI, Shenzhen, China) was used to carry out the double-enzyme digestion to construct six libraries, re-sequenced using the BGISEQ-500RS platform with an average 12× sequencing depth and coverage of 8%.

### Data Preparation and Statistical Analysis

#### Genotyping Data

To obtain better quality sequencing data, the raw data was filtered using the software SOAPnuke (Chen et al., 2018). The clean reads were then aligned with the *Anser cygnoides domesticus* genome data (https://www.ncbi.nlm.nih.gov/genome/31397?genome_assembly_id=229313) using BWA (Li and Durbin, 2010; Lu et al., 2015). The software SAMtools and GATK4 (https://software.broadinstitute.org/gatk/download/) then were used to detect variations and SNPs (Li, 2011). To limit the number of false positives and low confidence variants, all called variants were filtered using hard filters set according to the Broad Institute's hard filtering recommendations: quality by depth (QD) 2.0, read position rank sum −8.0, Fisher strand (FS) 60.0, root mean square (RMS) mapping quality (MQ) 40.0, strand odds ratio (SOR) 3.0, mapping quality rank sum test (MQ Rank Sum) −12.5, quality 30, minimum allele frequency 5%, call rate 70%, and Hardy–Weinberg equilibrium (HWE) *P* > 1e^−6^.

Then, Vcftools was used as a secondary filter, according to the following criteria: minor allele frequency (MAF) 0.05, HWE *P* = 1e^−6^, and max-missing 0.7 (Danecek et al., 2011).

Given the large number of scaffolds, scaffolds were combined into 21 chromosomes. The ordered SNP loci were separated into the 21 artificial chromosomes per 50 million base pairs (i.e. 1–50 Mbps, 51–100 Mbps etc.). Principal component analysis (PCA) was performed to identify genetic variation and the population structure.

#### Phenotypic Data

Descriptive statistics of phenotypic data were carried out by SPSS 22 software (IBM Corp., Armonk, NY, USA), and the sample size, maximum, minimum, average and standard deviation of high and low egg production samples were calculated.

#### Statistical Analysis

Genome wide association studies was performed using the EMMAX software with egg production character classified by dichotomies, i.e., the data was divided into high and low egg production (Kang et al., 2010). The analysis model was as follows:

P=μ+Zα+SNP+e

where P is the vector of phenotypes of the individuals, μ is the intercept of a straight line, Z is the incidence matrix of random polygenic effects, α is the random polygenic effects, SNP is the effect of a single nucleotide polymorphism, and e is the vector of residual errors with e ~ N (0, Iσ_e_), where I is the identity of matrix and σ_e_ is the residual variance.

#### Multiple Hypothesis Testing and Correction

The tested SNP markers could be used to make a Bonferroni adjustment with the 5% GWAS-wide significance level:

$$\text{α}=\frac{0.05}{\text{n}}$$

where α is the GWAS-wide significance level, n is the number of all tested SNP sites. In order to reduce false negative, and then extending the threshold 20 times as the suggestive value.

#### Population Stratification

Population stratification refers to the existence of subpopulations with different allele frequencies, which may pose a great threat to the validity of GWAS results, and even leads to false-positive results. The quantile–quantile plot (Q-Q plot) was used to assess the GWAS results, to judge whether the *P*-value calculated by SNP correlation analysis deviated from the hypothesis test on the whole overall.

### Detection of Candidate Genes

Based on the NCBI database (http://www.ncbi.nlm.nih.gov/) and Ensemble (http://www.ensemblgenomes.org), these SNPs identified by GATK4 were located in or near 30 genes.

### Quantification of Candidate Genes

To observe whether the candidate genes were differentially expressed in the high egg production group compared with the low group, we performed quantitative real-time polymerase chain reaction (RT-qPCR) for these genes. Total RNA was extracted from PBCs using the TRIzol reagent, and synthesized into cDNA using a Reverse Transcription Kit (Takara, Shiga, Japan). The cDNA was then used as a template for RT-qPCR using the CFX96 Touch (Bio-Rad, Hercules, CA, USA). The RT-qPCR primer sequences were synthesized by Sangon Biotech (Guangzhou, China) and were stored at −20°C for later use. According to the instructions of 2× SYBR Green qPCR Master Mix kit (Bimake, Houston, TX, USA), the RT-qPCR reaction was performed in triplicate and uses comprises 20 μl, containing 10 μl of 2× SYBR Green qPCR Master Mix, 0.4 μl of ROX Reference Dye, 1 μl of cDNA template, and a 0.5 mM concentration of specific primers. Thermal cycling parameters were as follows: 95°C for 5 min; 40 cycles of 95°C for 15 s, 60°C for 30 s, and 72°C for 30 s and 1 cycle of 95°C for 15 s, 60°C for 60 s, and 95°C for 15 s. Relative mRNA expression levels were calculated using the 2^−ΔΔCt^ method and normalized using the expression of GAPDH [encoding glyceraldehyde-3-phosphate dehydrogenase, (Livak and Schmittgen, 2001)]. All the primers for RT-qPCR are shown in **Table S1**.

## Results

### Sample Phenotypic Data Statistics

The egg production performance of lion head goose was divided into a high production group (>35 eggs per year) and a low production group (<25 eggs per year).

**Figure 1:**
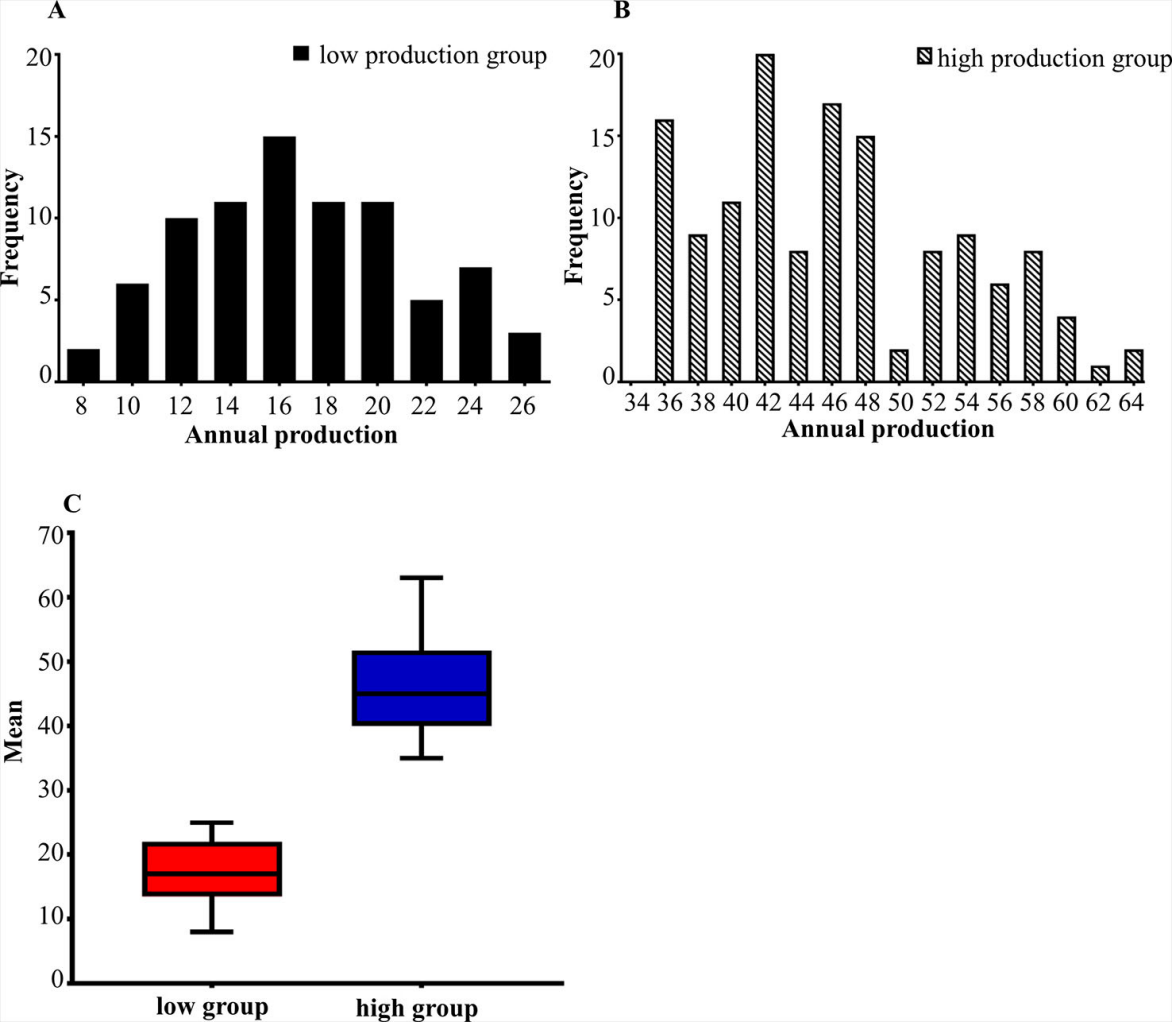
**(A, B)** The distribution of egg production in two groups. The horizontal axis shows the annual egg production and the vertical axis shows the frequency. **(A)** is the distribution of low egg production group. **(B)** is the distribution of high egg production group. **(C)** is the boxplot of the average between the high and low egg production groups.

The phenotypic data of low egg production was graphically recorded in **Figure 1A** and high egg production was in **Figure 1B**. The sample size, maximum, minimum, average and standard deviation of the trait measured in the current experiment were presented in **Table 1** and the boxplot is shown in **Figure 1C**. The sample size, maximum, minimum, average and standard deviation of the high egg production group were 136, 63, 35, 46, 54, while that in the low egg production group were 81, 25, 8, 17, 21, respectively. The annual egg production records for each individual are shown in **Table S2**.

**Table 1:** Phenotypic statistics.

Group	Sample size	Maximum	Minimum	Average	Standard deviation
Low group	81	25	8	17	21
High group	136	63	35	46	54

### Sequencing Data Statistics

Aligning the clean reads to the reference sequence allowed us to statistically analyze the sequencing depth, coverage rate, mapping rate, and mismatch rate, as shown in **Table 2**. Based on 217 original high egg production and low egg production samples, 8 were excluded because of mismatch, leaving 209 samples (131 high egg production, 78 low egg production). And the average of sequencing depth, coverage rate, mapping rate, and mismatch rate are 12.05%, 7.56%, 91.31%, and 1.48%, respectively.

**Table 2:** Sequencing statistics.

	Depth	Coverage (%)	Mapping rate (%)	Mismatch (%)
Min	1.26	0.95	88.10	1.20
Max	35.64	16.85	93.06	1.79
Mean	12.05	7.56	91.31	1.48

### Genetic Variation and Population Structure

**Figure 2:**
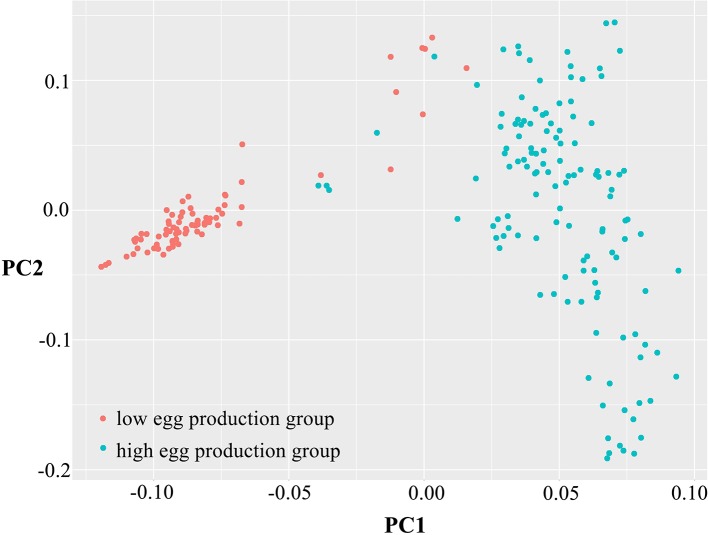
Principal component analysis of egg production. Principal component 1 (PC1) and principal component 2 (PC2) values comprised the X-axis and the Y-axis and were used to draw the scatter gram, and each dot represents one sample. Red points represent low-yield samples and blue points represent high-yield samples.

To determine data validity and population structure, PCA was performed based on the variation of the sequence data, taking principal component 1 as the horizontal and principal component 2 as the ordinate (**Figure 2**). The differences among individuals in each group were small, having high similarity. However, the dispersion between the high and low egg production groups was large, showing obvious population differentiation and indicating that there was a great difference between the two groups.

### Significant Single-Nucleotide Polymorphisms and Population Stratification Assessment

**Figure 3:**
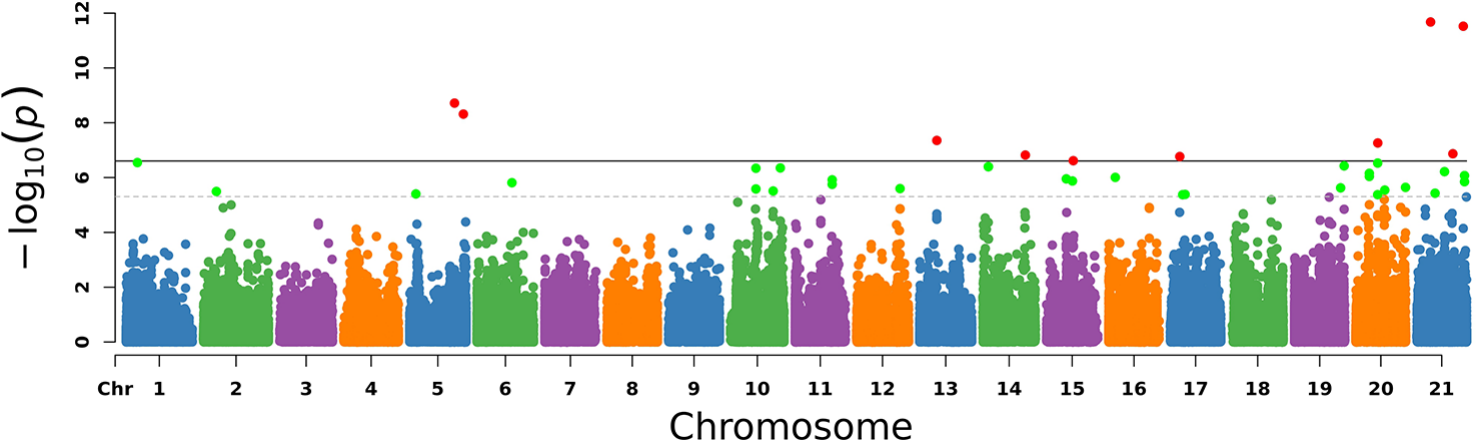
Manhattan plot of –log10 (*P*-values) for the egg laying traits in chromosome order. Each simulated chromosome contains 50 million bases. The solid line indicates the 5% significance level and the dotted line indicates the suggested level that extended the threshold to 20 times. The red points and green points are the significant SNPs.

The PCA results were used as covariates and EMMAX was used for the GWAS analysis. In **Figure 3**, chromosomes 1–21 are shown separately with different colors. The corresponding horizontal lines indicated the 5% GWAS-wide significance levels and the threshold was expanded 20 times as a second suggested value. The results are shown in **Figure 3**.

With the conditions of QD 2.0, Read Pos Rank Sum −8.0, FS 60.0, MQ 40.0, SOR 3.0, MQ Rank Sum −12.5, quality 30, minimum allele frequency 5%, call rate 70%, and HWE *P* > 1e^−6^, we finally identified 30 significant SNPs and the genes located on or near them, as shown in **Table 3**.

**Table 3:** The SNPs related to egg laying trait was detected by GWAS.

Number	SNP	Position	Gene	*P* value
1	NW_013185654	11050658	*CDH23*	2.84E−07
2	NW_013185657	12830867	*HSD17B12*	3.21E−06
3	NW_013185670	6211741	*EPHB3*	3.95E−06
4	NW_013185674	6277159	*GM2A*	1.92E−09
5	NW_013185675	5989069	*GNA12*	4.83E−09
6	NW_013185680	1530955	*NEXN*	1.54E−06
7	NW_013185711	3613832	*GPC4*	4.56E−07
8	NW_013185714	4862813	*FRMPD3*	3.09E−06
9	NW_013185716	1472974	*NFYC*	4.45E−07
10	NW_013185724	1493289	*GPC3*	1.77E−06
11	NW_013185736	3684650	*HTF3A*	2.53E−06
12	NW_013185743	1607400	*FGF9*	4.42E−08
13	NW_013185754	2051719	*FRY*	4E−07
14	NW_013185766	1522744	*ANTXR*	1.51E−07
15	NW_013185777	85282	*CLPB*	1.12E−06
16	NW_013185779	779290	*SMG7*	1.30E−06
17	NW_013185791	1229858	*SERPINC1*	9.85E−07
18	NW_013185814	703561	*SLITRK6*	1.71E−07
19	NW_013185815	1430965	*BMP4*	4.21E−06
20	NW_013185816	1738455	*RXRA*	4.12E−06
21	NW_013185899	252542	*CRTC1*	2.37E−06
22	NW_013185902	401032	*KCNAB2*	3.75E−07
23	NW_013185915	928804	*TMLHE*	9.1E−07
24	NW_013185925	301227	*LIMA1*	5.46E−08
25	NW_013185930	275521	*DDX49*	2.86E−06
26	NW_013185967	383783	*ELOVL4*	2.28E−06
27	NW_013186001	299910	*ZMAT5*	2.11E−12
28	NW_013186015	302128	*LIF*	3.73E−06
29	NW_013186054	67611	*FAAH2*	6.08E−07
30	NW_013186105	38036	*FBXL20*	1.35E−07

GWAS, genome-wide association studies; SNPs, single-nucleotide polymorphisms.

**Figure 4:**
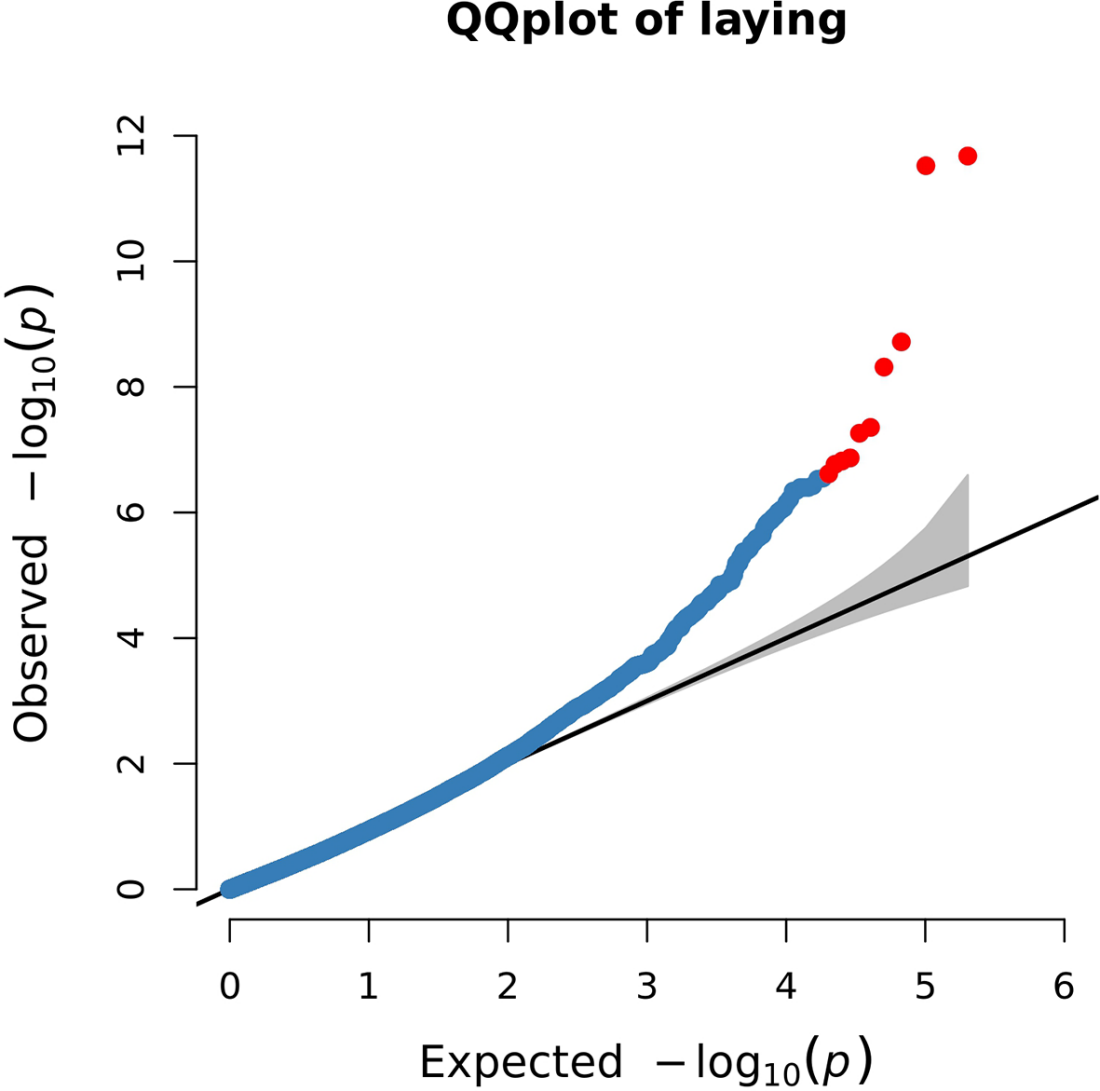
Quantile–quantile (Q-Q) plot of genome-wide association results for egg production. The blue points represent SNPs, and the red points represent the most significant SNPs.

The Q-Q plot showed that the screened SNPs were located above the diagonal line, indicating that the analytical model is reasonable. And the significantly higher points located at the top right corner of the graph represented potential candidate molecular markers associated with the trait (**Figure 4**).

### Candidate Genes Analysis

**Figure 5:**
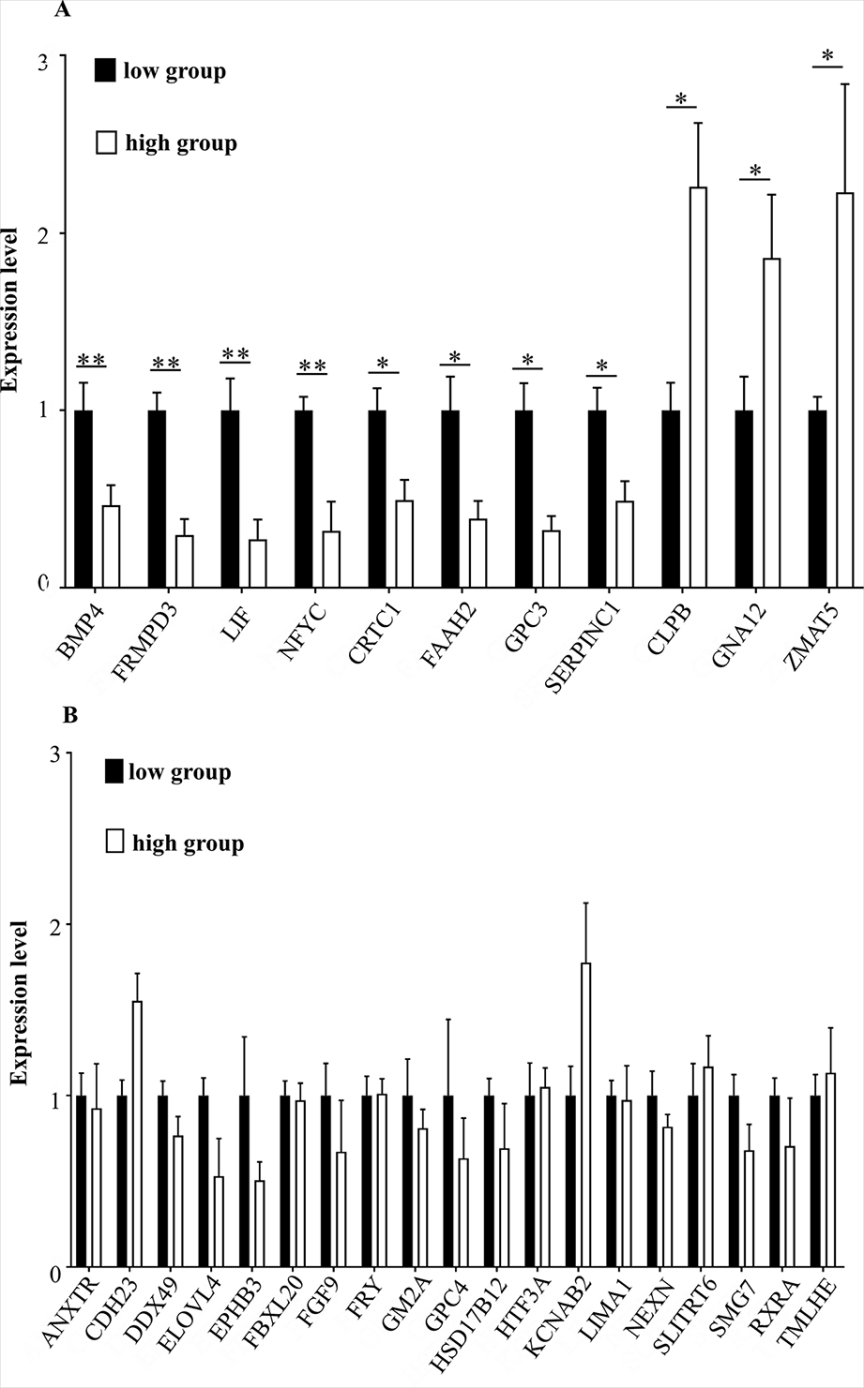
**(A, B)** The mRNA expression levels of key genes in different groups. The horizontal axis shows the different genes, and mRNA expression levels are on the vertical axis. Sign ** indicates extreme significance (*P* < 0.01). Sign * indicates significance (*P* < 0.05). No marker means no difference.

To determine whether the candidate genes were differentially expressed in the high and the low egg production group, we performed RT-qPCR on these genes. The expression levels of *BMP4* (encoding bone morphogenetic protein 4), *FRMPD3* (encoding *FERM* and *PDZ* domain containing 3), *LIF* (encoding Leukemia inhibitory factor), and *NFYC* (nuclear transcription factor Y subunit gamma) in the high egg production population were significantly lower than those in the low egg production population (**P < 0.01). The expression levels of *CRTC1* (encoding *CREB*-regulated transcription coactivator 1), *FAAH2* (encoding fatty acid amide hydrolase 2), *GPC3* (encoding glypican 3), and *SERPINC1* (encoding serpin family C member 1) in the high egg production population were significantly lower than those in the low egg production population (*P < 0.05). The expression levels of *CLPB* (encoding caseinolytic peptidase B protein homolog), *GNA12* (encoding guanine nucleotide-binding protein subunit alpha-12), and *ZMAT5* (encoding zinc finger, matrin type 5) in the high egg production population were significantly higher than those in the low egg production population (*P < 0.05, **Figure 5A**). The expression levels of the remaining genes (see **Figure 5B** for their symbols) were not significantly different between the two groups (**Figure 5B**).

## Discussion

### The Significance of Studying the Lion Head Goose

The lion head goose is the largest meat goose currently bred in China. It is characterized by a large size, crude feed tolerance, fast growth, high forage reward, strong stress resistance, and has a delicious meat that has extremely high economic value and is deeply favored by consumers (Zhuang and Lin, 2006). Therefore, an in-depth study of the breeding problem of lion head geese will help to modernize the industry to meet market demand.

In this study, the high and low egg production populations selected in a previous study were analyzed, and the number of eggs per year was used as the parameter to carry out GWAS. The phenotypic records of the egg laying character in this research were normally distributed, which was consistent with the separation characteristics and population separation characteristics.

### Application of Genome-Wide Association Study

In this study, we performed a GWAS for the egg production trait of a lion head geese population. Genomic studies have been carried out for many agricultural animals, such as chickens, swine, sheep, cattle, and geese, however, few of them have studied regionally important economic species such as lion head geese in China. To the best of our knowledge, this is the first GWA studies for the egg production character of lion head geese. Currently, database such as NCBI and Ensembl contain few reported goose sequences, which need to be further verification.

The research on the breeding of lion head geese has lagged behind that for other economically important species, which has led to many problems in the lion head goose breeding industry, such as backward breeding, and low productivity. With the development of genome-enhanced breeding and the improved the efficiency of genomic selection, it will be possible to protect and develop the breeding resources of the lion head goose, thus promoting the modernization and industrialization of the lion head goose industry.

### Significance of This Research

Next generation sequencing technology based on high-throughput sequencing and molecular marker technology, enables the fine mapping of functional genes. Genome selection technology represents a new generation of molecular breeding technology for livestock and poultry. This technique has been successfully applied to the cultivation of sheep (Zhao and Zhang, 2019). The high egg production traits of the lion head goose breeding population, the identification of the SNPs, and the selection of functional genes for economic traits will lay the foundation for the development of genotyping technology for lion head goose breeding.

### Detection and Verification of Key Genes

Bioinformatic analyses at the Ensembl and NCBI databases were used to identify the genomic location of SNPs that are significantly associated with the selected trait. Subsequently, bioinformatics and comparative genomics analysis were used to select key genes and make preliminary annotations on related gene functions.

In this study, a GWAS was conducted on the egg production trait of lion head geese, which detected 30 SNPs that were significantly associated with the high egg-production characteristic of lion head geese. We then screened the 30 genes that contained or were near, the SNPs.

Genes *BMP4*, *FRMPD3*, *LIF*, *NFYC*, *CRTC1*, *FAAH2*, *GPC3*, *SERPINC1*, *CLPB*, *GNA12*, and *ZMAT5* showed differential expression in between the high and low egg production populations of lion head geese.

In the present study, the expression levels of the *BMP4*, *LIF*, *NFYC* and *FRMPD3* genes in the low egg production population of the lion head goose were significantly higher than those in the high egg production population.

*BMP4* (bone morphogenetic protein 4), a member of the transforming growth factor beta (TGFβ) superfamily of growth factors, was first characterized for its role in bone metabolism (Nilsson and Skinner, 2003). It was subsequently reported to be involved in the regulation of embryonic mesoderm formation, and the formation of primordial germ cells (Nilsson and Skinner, 2003). *BMP4* mediates the formation of the mesoderm in mouse embryos, in which knockdown of *BMP4* leads to death and neonatal malformation (Zhu et al., 2002). It was reported that *BMP4* inhibits secretion of progesterone by granulosa cells and the expression of follicles in sheep and cattle (Monget et al., 2002; Da et al., 2018). Our results were consistent with these reports, i.e., *BMP4* might negatively affect the egg production character of the lion head geese.

*FRMPD3* (*FERM* and *PDZ* domain containing 3), located on the human X chromosome, is homologous to *FRMPD4*, which indicated that *FRMPD3* might mediate significant functions related to excitability associated with neuronal migration abnormality; however, the functions of *FRMPD3* have not been reported to be associated with poultry laying performance (Mardinly, 2013).

*LIF* (leukemia inhibitory factor), a secretory glycoprotein, is essential for the embryo implantation process in mice and humans (Aghajanova, 2004). Females lacking *LIF* are infertile, because their blastocysts cannot be implanted in the uterus, resulting in no clinical pregnancy (Steck et al., 2004). Our results would seem to conflict with those of previous reports, might reflect species inconsistency, or other, as yet unidentified factors. This result requires further verification and testing (Schofield and Kimber, 2005).

*NFYC* (nuclear transcription factor Y subunit gamma), a histone-fold domain-containing transcription factor, was identified in mice and humans as an oncogene required for the initiation and progression of tumors, and it engaged in chromatin remodeling (Tong et al., 2015). As far as we know, it has never been linked to reproductive function in any species.

The expression levels of the *CRTC1*, *FAAH2*, *GPC3*, and *SERPINC1* genes in the high egg production goose population were significantly lower than those in the low egg production population.

*CRTC1* (*CREB*-regulated transcription coactivator 1) is a transcriptional coactivator that has a biological function that affects energy balance and reproduction. Overexpression of *CRTC1* in mice led to obesity and infertility (Altarejos et al., 2008). Breuillaud *et al.* showed that the *CREB* coactivator *CRTC1* is indispensable for mouse fertility (Breuillaud et al., 2009). Our results were consistent with these reports, suggesting that *CRTC1* plays a negative role in the laying trait of the lion head goose.

*FAAH2* (fatty acid amide hydrolase 2), a member of the serine hydrolase family of enzymes, regulates several physiological processes, including appetite, inflammation, and various reproductive processes like secretion of gonadotropin-releasing hormone from the hypothalamus (Lunetta et al., 2015). *FAAH2* may participate in negative regulation of egg laying.

*GPC3* (glypican 3), a member of the heparan sulfate proteoglycans, has been widely studied as a target in human cancer, such as ovarian carcinoma. *GPC3* mediates the synthesis of integral membrane proteins that interact directly with insulin like growth factor 2 (*IGF2*), which is considered to be an important growth factor in ovarian cancer (Ofuji et al., 2014; Wu et al., 2016). *GPC3* induces apoptosis in ovarian cells, suggesting that it plays an important role in the development of ovarian cancer (Gonzalez et al., 1998). According to comprehensive research reports, we believe that low expression of *GPC3* may promote egg laying in the lion head goose.

*SERPINC1* (serpin family C member 1), is the main endogenous anticoagulant. Its mutations cause hereditary antithrombin deficiency and are associated with increased risk for all forms of pregnancy-related complications, which cause adverse pregnancy reaction (De la Morena-Barrio et al., 2012; Rogenhofer et al., 2014). Thus, *SERPINC1* may encourage low egg production; however, its specific effects require to be further verified.

The expression levels of *CLPB*, *GNA12*, and *ZMAT5* in the high egg production population were significantly increased compared with those in the low egg production population.

*CLPB* (caseinolytic peptidase B protein homolog) encodes an ATP-dependent chaperone. Disruption of *CLPB* is related to human congenital microcephaly and small birth weight (Capo-Chichi et al., 2015). Our results hint at a similar effect in geese. An increase in *CLPB* might lead to an increase in egg laying.

*GNA12* (guanine nucleotide-binding protein subunit alpha-12), the α subunit of a heterotrimeric G protein, participates in cell transformation and embryonic development; is expressed in the cytoplasm of Leydig cells; and has the biological function of promoting the differentiation of cells and elongated sperm cells into mature sperm (Hu et al., 2008, Udayappan and Casey, 2017). Shen et al. showed that preeclampsia is associated with decreased methylation of GNA12 promoters (Shen et al., 2015). Thus, the expression of *GNA12* might promote high egg production in the lion head goose.

For *ZMAT5* (zinc finger, matrin type 5), there have been no reports of its effects on animal reproduction.

## Conclusions

In this study, based on the breeding group of lion head goose, the blood DNA samples were collected to conduct a genome-wide association study on egg production traits. Thirty SNPs related to egg-producing traits were identified, and thirty genes located in or near SNPs were screened. The selected key genes were verified using RT-qPCR. The *BMP4*, CRTC1, *FAAH2*, *FRMPD3*, *GPC3*, *LIF*, *NFYC*, and *SERPINC1* genes might play a negative role in the egg production character of the lion head Goose. The *CLPB*, *GNA12*, and *ZMAT5* genes might play a positive role in egg production character in the laying trait of the lion head goose. The *ANTXR*, *CDH23*, *DDX49*, *ELOVL4*, *EPHB3*, *FBXL20*, *FGF9*, *FRY*, *GM2A*, *GPC4*, *HSD17B12*, *HTF3A*, *KCNAB2*, *LIMA1*, *NEXN*, *SLITRT6*, *SMG7*, *RXRA*, and *TMLHE* genes might have no significant effect on egg production character of the lion head goose. These results require further verification and confirmation.

In the past few years, GWASs have devoted to the identification of key loci and genes related to the molecular breeding of livestock and poultry. These genes may provide novel target for hereditary approaches to improve breeding. Developments in this area will be exciting and will affect the future of genomic breeding. In view of the fact that most of these genetic connections are limited, a large number of sample studies are required in future investigations in order to detect these subtle variations.

## Data Availability Statement

The datasets PRJNA552198 for this study can be found in the NCBI (https://www.ncbi.nlm.nih.gov/bioproject/PRJNA552198).

## Ethics Statement

The use of animals in this study was approved by the South China Agricultural University Committee for Animal Experiments (approval ID: SYXK(Guangdong)2019-0136). All study procedures and animal care activities were conducted in accordance with the national and institutional guidelines for the care and use of laboratory animals. Written informed consent was obtained from the owners for the participation of their animals in this study.

## Author Contributions

QZ and QX are the principal investigator for this article and contributed to the concept and planning of the article, collection of the data, and reporting of the work described. QZ, JC, XZ, ZL, and QX contributed to the planning of the article, collection of the data, and reporting of the work described. ZX, HL, and WL are the other principal investigators for this article and contributed to the concept of the manuscript, planning of the article, collection of the data, and reporting of the work described. All authors contributed to drafting the article or revising it critically for important intellectual content.

## Funding

This work was supported by the National Modern Agricultural Industry Science and Technology Innovation Center in Guangzhou (2018kczx01) and the Agricultural Science and Technology Innovation and Promotion Project in Guangdong Province (2018LM1112). The funders had no role in the study design, data collection and analysis, decision to publish, or preparation of the manuscript.

## Conflict of Interest

The authors declare that the research was conducted in the absence of any commercial or financial relationships that could be construed as a potential conflict of interest.
