# Supplementary material for: Genome-Wide Association Analysis Reveals Key Genes Responsible for Egg Production of Lion Head Goose
Source: Front Genet. 2020 Jan 28;10:1391. doi: 10.3389/fgene.2019.01391 (PMC6997537; doi:10.3389/fgene.2019.01391)
Supplement: Supplementary file 1 [file DataSheet_1.docx]

**Supplementary Materials**

| **Table S1 \|** Sequences of primer used in the study. | | | |
| --- | --- | --- | --- |
| **Gene** | **Forward Primer Sequence** | **Reverse Primer Sequence** | **Length(bp)** |
| *ANTXR2* | 5′-AACAAGGAGACGAGGAACGG-3′ | 5′-AGTAAGGAACGTGAACGCCA-3′ | 109 |
| *BMP4* | 5′-TCCGCTTCGTCTTCAACCTC-3′ | 5′-TGTTTATCCGGTGGAAGCCC-3′ | 129 |
| *CDH23* | 5′-GGACCCTCTTGGGGAGTTTG-3′ | 5′-AGGTTCATCGGCAGGTTGAG-3′ | 216 |
| *CLPB* | 5′-TTATTGCTGGAAGGCACGGA-3′ | 5′-TAGCTGCAAGCAGGGTCTTC-3′ | 112 |
| *CRTC1* | 5′-AAATCGTTGGCTGCTGCTTG-3′ | 5’-AGCAGAGTCAAAACCGCAGA-3’ | 140 |
| *DDX49* | 5′-CTCAACTCCGACTACAGCCC-3′ | 5′-CCGAGGATGAAACCCGAACA-3′ | 139 |
| *DKC1* | 5′-GCGTGGTGGCAAAGATCAAG-3′ | 5′-GGAATCTGGTGTGCTCTCGT-3′ | 152 |
| *ELOVL4* | 5′-GATGATTGGCCCTTGATGCAG-3′ | 5′-ACTGGAAGGGCTCTCTTGTCT-3′ | 183 |
| *EPHB3* | 5′-CAACCAGGTTCAGTTTGCGG-3′ | 5′-TTCAAACCGATGAGCACCGA-3′ | 264 |
| *FAAH2* | 5′-GCCATAAACCCACCACAGGA-3′ | 5′-CCAGCGACACCTTTTCGTTC-3′ | 183 |
| *FBXL20* | 5′-TATGCAGAGGGTGCCACAAG-3′ | 5′-CGCACTCTTCCAGGTCCATT-3′ | 204 |
| *FGF9* | 5′-CGACAATCTCTTTTGTGCCTGT-3′ | 5′-AATCCTCTGGGGGTAAGCTA-3′ | 107 |
| *FRMPD3* | 5′-TTCAGCGGTGTTCTCTGTCC-3′ | 5′-TGCATGGGGATGGAGTAGGA-3′ | 174 |
| *FRY* | 5′-CTGGTTCTGCTGTGTAGTT-3′ | 5′-ATCATAGGTCTGTCATCATCC-3′ | 119 |
| *GM2A* | 5′-CATCTGCACCATCCTCGACA-3′ | 5′-TAGTTGCCGTTGGTCATCCA-3′ | 123 |
| *GNA12* | 5′-AGGGAACCAGTTTGCTCACC-3′ | 5′-GAGCCTACAGAGCCAAACCA-3′ | 120 |
| *GPC3* | 5′- GAGGAAGATGGAAGAGAAGTA-3′ | 5′- CTTGGAAGATGGCAGCAT-3′ | 116 |
| *GPC4* | 5′-CAAGGGGATTTGGACACCGA-3′ | 5′-CTAAAGCGAGCGCTGAAACC-3′ | 257 |
| *HSD17B12* | 5′-GAAGCGTGGAATGAAGGT-3′ | 5′-AGGTCTGGAACATCAATGAA-3′ | 240 |
| *HTF3A* | 5′-TGCAGAACCTCAAGCCCATC-3′ | 5′-AGAGCGAGAGGTTGATGTCG-3′ | 169 |
| *KCNAB2* | 5′-CGACACAGCAGAGGTCTACG-3′ | 5′-ATCCCCTGGTTGATGACGTG-3′ | 204 |
| *LIF* | 5′-GCGAAGCTAAGGACAGCAGA-3′ | 5′-CAGCATCGGCAGGAGGTAAT-3′ | 179 |
| *LIMA1* | 5′-AAAGGGGGAATCTGAGCGTG-3′ | 5′-CTCCGGGTCTGAGCAAGAAG-3′ | 123 |
| *NEXN* | 5′-CCTTCCTCCCACCATTTCCAG-3′ | 5′-ACTTGCACAGCCTATCTTGACA-3′ | 165 |
| *NFYC* | 5′-ATGGAGGAGATCCGCAACCT-3′ | 5′-ATCCACGCTCGCAAAGTCAA-3′ | 169 |
| *RACGAP* | 5′-AGTAATGGCTCTTGCACCCC-3′ | 5′-GACACTCTGGATGAGCGACC-3′ | 169 |
| *RXRA* | 5′-CGTACATTGAAGCGAACAT-3′ | 5′-GGAGAAGGAGGCGATTAG-3′ | 200 |
| *SERPINC1* | 5′-TAGGCAAACGGGGGATCTGT-3′ | 5′-TGGATAAGGCTTGACAATCAGGA-3′ | 192 |
| *SLITRT6* | 5′-ACAATCCTGCGGTCCCTTAC-3′ | 5′-GCCGATGCAGAACAAGAACA-3′ | 121 |
| *SMG7* | 5′-AGTGTAGAGCAACGGAAGCC-3′ | 5′-CATGCCGGAGTCTCTAACCC-3′ | 139 |
| *TAF7L* | 5′-AGTCCTGGCGTAACTGTGTC-3′ | 5′-TTTCGATGATGCAGGGCAGA-3′ | 192 |
| *TMLHE* | 5′-TGCTACCTGACGAGAGACGA-3′ | 5′-GGTGTCTGAGGTACGTGCAA-3′ | 169 |
| *ZBTB16* | 5′-ACTTCTGCTGTTCTGGTCGG-3′ | 5′-CGCATCTGGTTGGCTTTGTG-3′ | 197 |
| *ZMAT5* | 5′-ACGACTTGTTCCGAGATGCT-3′ | 5′-GCAGTTGGACCCGAAATCAC-3′ | 104 |

| **Table S2 \|** Egg production records for each sample. | | | | | |
| --- | --- | --- | --- | --- | --- |
| **Number** | **Annual Production** | **Number** | **Annual Production** | **Number** | **Annual Production** |
| **Low egg production group** | | | | | |
| 01 | 8 | 28 | 14 | 55 | 18 |
| 02 | 8 | 29 | 14 | 56 | 19 |
| 03 | 9 | 30 | 15 | 57 | 19 |
| 04 | 9 | 31 | 15 | 58 | 19 |
| 05 | 9 | 32 | 15 | 59 | 19 |
| 06 | 10 | 33 | 15 | 60 | 19 |
| 07 | 10 | 34 | 15 | 61 | 19 |
| 08 | 10 | 35 | 15 | 62 | 19 |
| 09 | 11 | 36 | 15 | 63 | 20 |
| 10 | 11 | 37 | 16 | 64 | 20 |
| 11 | 11 | 38 | 16 | 65 | 20 |
| 12 | 11 | 39 | 16 | 66 | 20 |
| 13 | 11 | 40 | 16 | 67 | 22 |
| 14 | 12 | 41 | 16 | 68 | 22 |
| 15 | 12 | 42 | 16 | 69 | 22 |
| 16 | 12 | 43 | 16 | 70 | 22 |
| 17 | 12 | 44 | 16 | 71 | 22 |
| 18 | 12 | 45 | 17 | 72 | 23 |
| 19 | 13 | 46 | 17 | 73 | 23 |
| 20 | 13 | 47 | 17 | 74 | 23 |
| 21 | 13 | 48 | 17 | 75 | 24 |
| 22 | 13 | 49 | 17 | 76 | 24 |
| 23 | 13 | 50 | 17 | 77 | 24 |
| 24 | 13 | 51 | 17 | 78 | 24 |
| 25 | 14 | 52 | 18 | 79 | 25 |
| 26 | 14 | 53 | 18 | 80 | 25 |
| 27 | 14 | 54 | 18 | 81 | 25 |
| **High egg production group** | | | | | |
| 0001 | 35 | 0047 | 41 | 0093 | 48 |
| 0002 | 35 | 0048 | 41 | 0094 | 48 |
| 0003 | 35 | 0049 | 42 | 0095 | 48 |
| 0004 | 35 | 0050 | 42 | 0096 | 48 |
| 0005 | 35 | 0051 | 42 | 0097 | 50 |
| 0006 | 35 | 0052 | 42 | 0098 | 50 |
| 0007 | 35 | 0053 | 42 | 0099 | 51 |
| 0008 | 35 | 0054 | 42 | 0100 | 51 |
| 0009 | 36 | 0055 | 42 | 0101 | 51 |
| 0010 | 36 | 0056 | 42 | 0102 | 51 |
| 0011 | 36 | 0057 | 43 | 0103 | 52 |
| 0012 | 36 | 0058 | 43 | 0104 | 52 |
| 0013 | 36 | 0059 | 43 | 0105 | 52 |
| 0014 | 36 | 0060 | 43 | 0106 | 52 |
| 0015 | 36 | 0061 | 43 | 0107 | 53 |
| 0016 | 36 | 0062 | 43 | 0108 | 53 |
| 0017 | 37 | 0063 | 43 | 0109 | 53 |
| 0018 | 37 | 0064 | 43 | 0110 | 53 |
| 0019 | 37 | 0065 | 45 | 0111 | 54 |
| 0020 | 37 | 0066 | 45 | 0112 | 54 |
| 0021 | 38 | 0067 | 45 | 0113 | 54 |
| 0022 | 38 | 0068 | 45 | 0114 | 54 |
| 0023 | 38 | 0069 | 45 | 0115 | 54 |
| 0024 | 38 | 0070 | 45 | 0116 | 55 |
| 0025 | 38 | 0071 | 45 | 0117 | 55 |
| 0026 | 39 | 0072 | 45 | 0118 | 55 |
| 0027 | 39 | 0073 | 46 | 0119 | 55 |
| 0028 | 39 | 0074 | 46 | 0120 | 55 |
| 0029 | 39 | 0075 | 46 | 0121 | 55 |
| 0030 | 39 | 0076 | 46 | 0122 | 57 |
| 0031 | 40 | 0077 | 46 | 0123 | 57 |
| 0032 | 40 | 0078 | 46 | 0124 | 57 |
| 0033 | 40 | 0079 | 46 | 0125 | 57 |
| 0034 | 40 | 0080 | 46 | 0126 | 58 |
| 0035 | 40 | 0081 | 46 | 0127 | 58 |
| 0036 | 40 | 0082 | 47 | 0128 | 58 |
| 0037 | 41 | 0083 | 47 | 0129 | 58 |
| 0038 | 41 | 0084 | 47 | 0130 | 59 |
| 0039 | 41 | 0085 | 47 | 0131 | 59 |
| 0040 | 41 | 0086 | 47 | 0132 | 59 |
| 0041 | 41 | 0087 | 47 | 0133 | 60 |
| 0042 | 41 | 0088 | 47 | 0134 | 61 |
| 0043 | 41 | 0089 | 47 | 0135 | 63 |
| 0044 | 41 | 0090 | 47 | 0136 | 63 |
| 0045 | 41 | 0091 | 47 |  |  |
| 0046 | 41 | 0092 | 48 |  |  |
